# Rapid Isolation of Extracellular Vesicles from Cell Culture and Biological Fluids Using a Synthetic Peptide with Specific Affinity for Heat Shock Proteins

**DOI:** 10.1371/journal.pone.0110443

**Published:** 2014-10-17

**Authors:** Anirban Ghosh, Michelle Davey, Ian C. Chute, Steven G. Griffiths, Scott Lewis, Simi Chacko, David Barnett, Nicolas Crapoulet, Sébastien Fournier, Andrew Joy, Michelle C. Caissie, Amanda D. Ferguson, Melissa Daigle, M. Vicki Meli, Stephen M. Lewis, Rodney J. Ouellette

**Affiliations:** 1 Atlantic Cancer Research Institute, Moncton, New Brunswick, Canada; 2 Department of Chemistry and Biochemistry, Université de Moncton, Moncton, New Brunswick, Canada; 3 New England Peptide Inc., Gardner, Massachusetts, United States of America; 4 Department of Chemistry and Biochemistry, Mount Allison University, Sackville, New Brunswick, Canada; 5 Department of Microbiology and Immunology, Dalhousie University, Halifax, Nova Scotia, Canada; 6 Department of Biology, University of New Brunswick, Saint John, New Brunswick, Canada; University of Cincinnati, College of Medicine, United States of America

## Abstract

Recent studies indicate that extracellular vesicles are an important source material for many clinical applications, including minimally-invasive disease diagnosis. However, challenges for rapid and simple extracellular vesicle collection have hindered their application. We have developed and validated a novel class of peptides (which we named venceremin, or Vn) that exhibit nucleotide-independent specific affinity for canonical heat shock proteins. The Vn peptides were validated to specifically and efficiently capture HSP-containing extracellular vesicles from cell culture growth media, plasma, and urine by electron microscopy, atomic force microscopy, sequencing of nucleic acid cargo, proteomic profiling, immunoblotting, and nanoparticle tracking analysis. All of these analyses confirmed the material captured by the Vn peptides was comparable to those purified by the standard ultracentrifugation method. We show that the Vn peptides are a useful tool for the rapid isolation of extracellular vesicles using standard laboratory equipment. Moreover, the Vn peptides are adaptable to diverse platforms and therefore represent an excellent solution to the challenge of extracellular vesicle isolation for research and clinical applications.

## Introduction

Heat shock proteins (HSPs) are one of the most ancient molecular defense systems. In non-stressed and non-transformed cells, HSPs are ubiquitously expressed in low amounts as intracellular proteins that exhibit various cytoprotective functions, including buffering the cell from stressful conditions, monitoring proper protein folding (chaperones), cellular housekeeping (proteasomes), and presenting antigens to immune cells [1], [2]. However, the cytoprotective effects of HSPs are also exploited by transformed cells to promote their own survival. In stressed and cancer cells, intracellular HSP-peptide complexes induce anti-apoptotic effects and act as cytoprotectants by directing damaged proteins for degradation, whereas extracellular HSPs elicit immune responses by carrying a variety of immunogenic peptides [3], [4].

Although intracellular chaperones/HSPs have been studied for the last five decades, studies of extracellular HSPs have only begun in recent years. The release of HSPs into the extracellular milieu is emerging as a characteristic of many pathological conditions, including infection and cancer. Recent studies have shown that a broad range of HSP paralogues that are normally restricted to discrete intracellular compartments are relocated to the surface of cancer and infected cells [5]–[7]. Importantly, the presence of HSPs on the surface of cancer and infected cells is a trait that is not shared by their normal counterparts. Hsp70 is an integral component of the cancer cell membrane *via* its affinity for phosphatidyl serine in the external membrane layer and the glycosphingolipid Gb3 in signaling platforms known as lipid rafts, despite the absence of an externalizing sequence [8]. In addition, exosome/extracellular vesicle-associated extracellular transport of HSPs is evident in many pathological conditions, including cancer [9]–[15].

Extracellular vesicles (EVs) are a heterogeneous population, both in size and in content, of nano-sized organelles released by most cell types. EVs contain an active cargo of molecules that represent the state of their cell of origin. The release of EVs is a conserved physiological process observed both *in vitro* and *in vivo.* EVs are found in a wide range of biological fluids, including blood, urine, saliva, amniotic fluid, and pleural fluid [16]–[22]. There are two main groups of extracellular vesicles: exosomes of endosomal origin (40–100 nm in diameter) and shed vesicles (or ectosomes) pinched off from the plasma membrane (50–1000 nm in diameter). We will refer to the collective group as EVs [23]. Pathological conditions, such as cancer, affect the amount and localization of EV protein content. Along with the HSPs, exosomal and EV protein markers include Alix, TSG101, the tetraspanins CD63, CD81, and CD9, HSPs, metalloproteinases, integrins, some glycoproteins, and selectins [24].

We set out to design synthetic peptides that specifically bind to HSPs. The peptide (substrate) binding domain of HSPs is well characterized, especially for Hsp70. In the Hsp70 protein family the substrate binding domain-β (SBD-β) in the C-terminal region forms a hydrophobic binding pocket to bind to substrate peptides or their partner co-chaperones. The well-characterized signature domain of substrate peptides to which the Hsp70 SBD-β binds is called the J-domain. J-domain-containing proteins constitute a conserved family of co-chaperones found in *E.coli* (DnaJ) and humans (Hsp40 and Hsj1) that bind with their partner chaperone, known as a DnaK homologue or Hsc70 respectively [25]–[27]. The J-domain consists of a four-bundle α-helix, where helices I and IV form the base and helices II and III form a finger-like projection of the structure. A conserved amino acid sequence, HPD (His-Pro-Asp), is located at the tip of the projection [28]. Many structural studies have indicated that the positively charged and hydrophobic amino acid residues of helix II and the HPD sequences of J-domains interact with the hydrophobic peptide binding domain (SBD-β) of the C-terminal parts of HSP70s [17], [28]–[33]. Based on these structural studies of the peptide binding pockets of Hsp70 [25]–[27], [34] we rationalized that: (1) an ideal HSP-binding peptide would be strongly cationic with hydrophobic side chains, consistent with properties conducive to stable association with the peptide binding cleft of Hsp70 isoforms and paralogues and (2) the avidity of those peptides with HSP-binding properties could be screened by counter migration during isoelectric focusing (IEF).

Accordingly, we designed and synthesized a series of peptides (that we collectively named venceremins, or Vn peptides), which were screened for their HSP-binding properties using IEF. Many tested peptides bound HSPs, but during the course of our experiments we discovered that at least one Vn peptide (Vn96) also precipitated small subcellular structures that resemble membrane structures of ER-Golgi origin at low centrifugal speed (10,000×g). These results prompted us to examine the potential of Vn96 as an exosome/EV capture tool from cell culture growth media and biological fluids.

## Materials and Methods

### Peptides

All the peptides were synthesized at New England peptide (Gardner, US). The Vn96, Vn20 peptides and their use to isolate EVs are patent pending (US 13/824,829. PCT number, PCT/CA2012/050175).

### Cell culture and cell lines

Breast cancer cell lines (MCF-10A, MCF-7 and MDB-MB-231) were purchased from the American Tissue and Culture Collection (ATCC) and grown in tissue culture according to the supplier’s recommended protocols. The cells were grown to 80–90% confluency, washed four times with serum-free media, and then incubated with a minimal volume of serum-free media required to cover the cells. After four hours of incubation the ‘conditioned’ cell culture media was collected, followed by removal of cellular material by a two-step centrifugation process (1,000×g and 17,000×g) and/or by filtering with 0.22 µm filters to remove large protein aggregates and other cellular debris. We then precipitated EVs from the collected conditioned cell culture media using either Vn96 peptides or a scrambled version of the Vn96 peptide as a negative control. The above cell lines were also adapted for continuous long-term conditioned cell culture media harvest in compartmentalized flasks (CELLine, AD 1000 bio-reactor) designed with a cell-growth chamber that is separated from the bulk cell culture media compartment with a 10 kDa cut-off dialysis membrane. The cell culture media added to the cell-growth chamber were prepared with exosome free (Exo-Free) Fetal Bovine Serum (FBS). FBS was purchased from Wisent Bioproducts (Quebec, Canada, Cat# 080–350). The Exo-free FBS was prepared by centrifugation of FBS at 100,000×g for two hours at 4°C followed by aspiration of the supernatant without disturbing the exosome pellet. The conditioned media were harvested once a week from the cell-growth chamber only. The harvested cell culture media were immediately centrifuged at 1,800×g for five minutes to remove the floating cells, followed by 17,000×g for 15 minutes to remove cellular debris; the prepared material was then stored at 4°C with 5 µl of protease inhibitor cocktail-III (EDM-Millipore) and 0.1% (v/v) ProClin300 (Sigma) as a preservative.

### Human sample collection and preparation

This study was reviewed and approved by the Vitalité Health Network Research Ethics Board (New Brunswick, Canada) prior to the beginning of sample collection. Written informed consent was obtained by a Clinical Research Associate from each patient before any blood or urine samples were collected. Whole blood (+EDTA) was collected from consenting healthy women and breast cancer patients. The plasma layer was collected after centrifugation of the whole-blood (EDTA) at 1,500×g for 15 minutes at room temperature (RT), followed by pre-clearing the plasma by centrifugation at 17,000×g at 4°C for 15 minutes. 5 µl of protease inhibitor cocktail-III and 0.1% (v/v) ProClin300 (preservative) were added to each millilitre of the pre-cleared plasma before archiving at 4°C (short-term) or −80°C (long-term) for storage. Urine samples were collected from consenting male patients scheduled for prostate biopsy subjected to both pre- and post-digital rectal examination (DRE) with prostate massage. The urine samples were centrifuged at 650×g for 10 minutes at RT; supernatants were collected and centrifuged again at 10,000×g for 15 minutes at RT, followed by a final centrifugation at 17,000×g for 15 minutes at RT. Aliquots of 7.5 ml were likewise archived at 4°C or −80°C with 7.5 µl each of protease inhibitor cocktail-III and ProClin300.

### EV isolation using Vn peptides

The archived conditioned cell culture media and corresponding ‘control’ media (unused) were cleared once again by centrifugation at 17,000×g following removal from the archive, and were then incubated with either: 1) biotinylated-Vn96 (b-Vn96) or biotinylated scrambled sequence of Vn96 (b-Scr-Vn96), or, 2) Vn96 or scrambled sequence of Vn96 (Scr-Vn96) overnight at 4°C (long incubation) or 15 minutes at RT (short incubation) with rotation. The peptides were used at either 100 µg/ml or 50 µg/ml of media. The incubated samples were centrifuged at 17,000×g at 4°C for 15 minutes or at 10,000×g for seven minutes at RT using a bench-top microcentrifuge for the long or short incubations, respectively. Semi-translucent precipitates were visible only in case of Vn96 and b-Vn96 incubated samples. All samples were washed three times with phosphate buffered saline (PBS).

The archived plasma samples were thawed and diluted 5 to 10 times with PBS, while the archived urine samples were thawed and used without dilution. The samples were subjected to clearing by centrifugation (17,000×g for 15 min at 4°C) and/or filtration though 0.2 µm pore-size filters. The cleared samples were incubated with 50 µg/ml Vn96 or Scr-Vn96 peptide overnight at 4°C with rotation, followed by precipitation by centrifugation at 17,000×g at 4°C for 15 minutes and three washes with PBS. The precipitated Vn96-EV complexes were processed for either electron microscopy, atomic force microscopy, RNA isolation, or proteomic analysis as described below.

### EV and exosome isolation using ultracentrifugation (UCF) and a commercially-available kit

We followed the protocol for EV and/or exosome preparation on a 30% sucrose cushion as described in the ‘Current Protocols in Cell Biology’ [41] with minor modifications. Briefly, approximately 10 ml of pre-cleared samples were transferred to UCF tubes (SW-40Ti rotors), followed by very careful insertion of a Pasteur pipette into the bottom of the sample in order to layer 500 to 750 µl of 30% sucrose solution in PBS at the bottom of the tube. The samples were centrifuged at 100,000×g for two hours. The exosome-containing sucrose cushions were aspirated carefully using a Pasteur pipette into a new ultracentrifuge tube, diluted to 10 ml with PBS and re-centrifuged at 100,000×g for 90 minutes. The supernatants were discarded and the exosome pellets were carefully resuspended in 50–100 µl of PBS with 5 µl of protease inhibitor. We used ExoQuick for the preparation of EVs from conditioned cell culture media following supplier’s instructions.

### Electron microscopy

The precipitated Vn96-EV complexes were incubated with 2 µg/ml proteinase K in PBS at 37°C for four hours to disperse the membrane-encapsulated EVs into solution, followed by centrifugation at 17,000×g for 15 minutes during which no visible pellet was observed. The dispersed EVs from the supernatants (5–10 µl) were deposited onto formvar/carbon-coated 200 mesh copper grids for 2–3 minutes, followed by floating on a 100 µl drop of water (on para-film) in a sample-side down orientation for one minute. Fixation was achieved with 3.7% formalin followed by two washes with water. The samples were contrasted with 2% uranyl acetate (w/v) to visualize membranes. The water, 3.7% formalin and 2% uranyl acetate were filtered through 10 kDa cut off filters before use on the EM-grids to remove any particulate contaminants. The dried grids were viewed using a JEOL 6400 electron microscope at the Microscopy and Microanalysis Facility, University of New Brunswick. Minimum three samples and technical repeats were performed to obtain the optimal concentration for visibility.

### Atomic force microscopy

Vn96-precipitated EVs were dispersed with proteinase K digestion in 50 µl PBS. The preparation was diluted 1∶100 in de-ionized water and adsorbed to freshly cleaved mica sheets that were rinsed with de-ionized water and dried under a gentle stream of nitrogen. Two to four biological repeats were used for each sample type. The samples were scanned in non-contact mode using a Park Systems XE-100 atomic force microscope equipped with a silicon cantilever (f0∼300 kHz, Park Systems). Topographic and phase images were recorded simultaneously at a resolution of 512×512 pixels, at a scan rate of 1 Hz. Image processing was performed using the Park Systems XEI software.

### Nanoparticle Tracking Analysis (NTA)

NTA is a method of size-distribution and concentration analysis of nano-particles in liquid, based on their sizes and Brownian motion using the Stokes-Einstein equation. We used NanoSight LM10 with NTA software (V2.3). The Vn96-EV complexes were dispersed by digestion with proteinase K in PBS as described above. UCF-prepared exosomes and Vn96-prepared, proteinase K-digested EVs were subjected to different PBS dilutions (0.1 µm filtered) to find the best windows for NTA video capture. The experiments were repeated at least four times to obtain representative results.

### Proteomic analysis

The EV-Vn96 complexes or UCF-purified exosomes were dissolved and heated for five minutes at 85oC in buffer (125 mM Tris pH 6.8 with 2% SDS) to harvest proteins for subsequent analysis. The protein samples were separated on SDS-PAGE and visualized with Coomassie EZBlue stain. Each entire lane was excised into several 2–3 mm long slices and distributed into different microcentrifuge tubes. Each band was treated with 10 mM dithiothreitol and 25 mM iodoacetic acid to reduce internal disulfide bonds and alkylate free cysteine resdues. Fifty microliters of a 20 ng/µL solution of trypsin was added to each band for overnight enzymatic cleavage.

Protein tryptic digest extracts were analyzed by gradient nanoLC-MS/MS using a Quadrupole Orbitrap (Q-Exactive, Thermo-Fisher Scientific) mass spectrometer interfaced to a Proxeon Easy Nano-LC II. Samples were adjusted to 1% aqueous acetic acid and injected (5 µL) onto a narrow bore (20 mm long×100 µm inner diameter) C18 pre-column packed with 5 µm ReproSil-Pur resin (Thermo-Fisher Scientific). High resolution chromatographic separation was then achieved on a Thermo-Scientific Easy C18 analytical column with dimensions of 100 mm by 75 µm i.d. using 3 µm diameter ReproSil-Pur particles. Peptide elution was achieved using an acetonitrile/water gradient system. LC-MS grade water and acetonitrile (EMD Millipore) were both obtained from VWR Canada (Mississauga, ON). Solvent A consisted of 0.1% formic acid in water and solvent B was made up of 90/9.9/0.1 acetonitrile/water/formic acid. Formic acid was purchased from Sigma-Aldrich Canada (Oakville, ON). A linear acetonitrile gradient was applied to the C18 column from 5–30% solvent B in 120 minutes followed by 100% B for 10 minutes at a flow rate of 300 nL/min.

The outlet of the nano-flow emitter on the Q-Exactive (15 µm diameter) was biased to +1.9 kV and positioned approximately 2 mm from the heated (250oC) transfer capillary. The S-lens of the mass spectrometer was maintained at 100 Volts. The Q-Exactive mass spectrometer was calibrated in positive ion mode with mass standards (caffeine, MRFA peptide and Ultramark) every three days as recommended by the instrument manufacturer. Mass spectrometric data was acquired in data dependent mode (DDA, data dependent acquisition) whereby a full mass scan from 350–1500 Th was followed by the acquisition of fragmentation spectra for the five most abundant precursor ions with intensities above a threshold of 20,000. Precursor ion spectra were collected at a resolution setting of 70,000 and an AGC (automatic gain control) value of 1×106. Peptide fragmentation was performed using high energy collision induced dissociation in the HCD cell and MS/MS spectra were collected in the Orbitrap at a resolution of 17,500 and an AGC setting of 1×105. Peptide precursors were selected using a repeat count of two and a dynamic exclusion period of 20 seconds.

Mass spectrometric protein identification data was analyzed using Proteome Discoverer version 1.3 (Thermo-Fisher Scientific) employing the Sequest scoring algorhithm. A human FASTA database was obtained from UniProt. Searches were performed with the following settings: (a) enzyme specificity of trypsin with two allowed missed cleavages, (b) precursor and fragment tolerances were 10 ppm and 0.8 Da, respectively, (c) a variable modification of methionine oxidation (+15.99 Da), and (d) a fixed modification of cysteine carboxymethylation (+58.00 Da). Proteome Discoverer 1.4 calculated a strict false discovery rate (FDR) of 0.1% based on the results of a decoy (reverse) database search. Proteins were assigned a positive identification if at least two peptides were identified with high confidence. Two biological samples were prepared for each sample type, and one representative dataset for each sample is presented here.

### Next-generation RNA sequencing

The conditioned cell culture media (from MCF-7 and MDA-MB-231 cells) were used to isolate EVs using the Vn96 peptide, ExoQuick and ultracentrifugation methods described above. RNA from the isolated EVs was harvested with TRIZOL reagent (Life Technologies) using a protocol adapted for small RNAs. Barcoded cDNA libraries were prepared using RNA-Seq Version 2 kit from Life Technologies following their recommended protocol. Library preparations were assayed for both quality control and quantity using Experion DNA 1K chip (Life Technologies) and diluted to 16 pM concentration. Samples were sequenced using a PGM Sequencer from Life Technologies on a 318 chip following the manufacturer recommended protocol. Each chip was loaded with three samples.

### Western-blot analysis

The purified Vn96-EV complexes and UCF-prepared exosomes were dissolved in 4x SDS-loading dye (with or without reducing agents). The proteins were resolved on either 10% or 4–12% gradient SDS-PAGE. The resolved proteins were transferred to either nitrocellulose or PVDF membranes followed by blocking and immunoblotting with indicated antibodies using chemiluminescence and other standard procedures. Each Western-blot experiment was performed at least four times. All the antibodies used were purchased from Santa Cruz Biotechnology.

## Results

### Selection and validation of HSP-binding peptides

**Figure 1:**
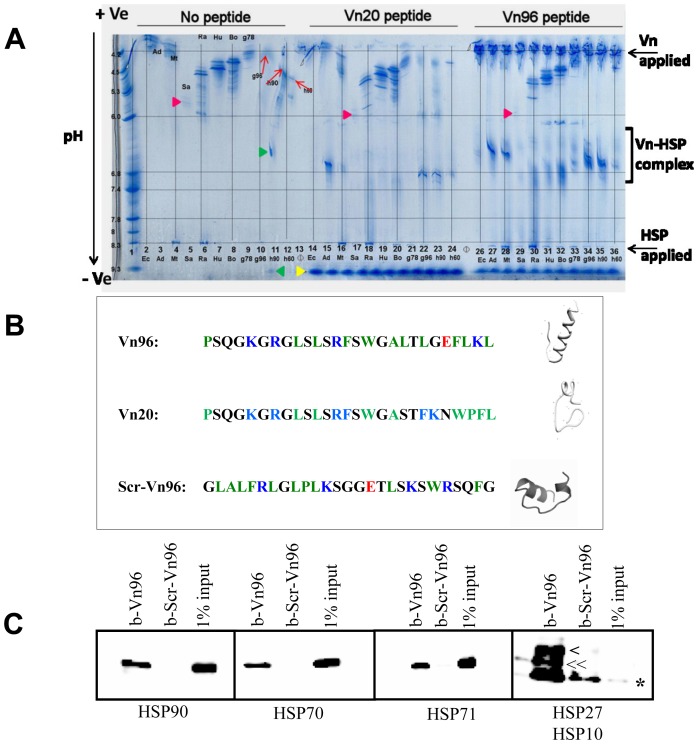
Selection and validation of peptides with HSP-binding properties. A. Peptide selection. Representative demonstration of peptide screening with recombinant HSPs using broad-range pH (3–10) isoelectric focusing (IEF) gels. Samples of 20 µg of the indicated peptides were applied at the anode and 2 µg of purified recombinant HSPs were applied at the cathode. The gradient of pH and electrophoretic directions are indicated on the left side of the gel. Abbreviations of recombinant HSP sources and horizontal lines are used to assist in sample identification in the distorted counter migrations affecting lanes 10–14. The complexes formed during counter migration are indicated at the right of the gel as “Vn-HSP complexes”. Estimates of the isoelectric focusing points of unbound gp96, HSP90 and HSP60 in the area of counter migrant distortion are indicated by red arrows (lane 10, 11 and 12). The yellow arrowhead at the bottom indicates unbound Vn peptide isoelectric focusing following counter migration against HSPs. Red arrowheads (lanes 5, 17 and 29) indicate the weakly staining salmon HSP70 (despite standardized dilution). The green arrowhead (middle of lane 11) indicates a complex with HSP90 resulting from Vn20 that has extended binding influence across preceding adjacent lanes. The green arrowhead at the base of the gel (lane 11) indicates the final focusing point of the errantly migrating Vn20. Abbreviations are as follows: Ec, *E.coli* dnaK (lanes 1, 14,26); Ad, *A.davidanieli* HSP70 (lanes 3, 15, 27); Mt, *M.tuberculosis* HSP70 (lanes 4, 16, 28); Sa, Chinook salmon HSP70 (lanes 5, 17, 29); Ra, rat HSP70 (lanes 6, 18, 30); Hu, human HSP70-1 (lanes 7, 19, 31); Bo, bovine HSP70-8 (8, 20, 32); gp78, hamster HSP70-5 (lane 9, 21, 33); gp96, canine GRP96 (lanes 10, 22, 34); h90, human HSP90 (lanes 11, 23, 35); h60, human HSP60 (lanes 12, 24, 36) and as blank lanes (lanes 13 and 25). B. Sequences of Vn96, Vn20 as well as Scrambled-Vn96 (Scr-Vn96) and their predicted 3D structures in aqueous solution using PEP-FOLD server [55]. Red, blue, green, and black amino acid residues are acidic, basic, hydrophobic uncharged and other amino acid residues, respectively. Note that the Vn96 peptide favors a helical conformation. C. Validation of HSP binding by the Vn96 peptide *via* affinity pull-down of HSPs from total cell lysate. MCF-7 breast cancer cells were lysed and processed as described in experimental procedures. Streptavidin-coupled magnetic beads saturated with either biotinylated-Vn96 (b-Vn96) or biotinylated-scrambled sequence of Vn96 (b-Vn96-Scr) peptides were used to perform the pull-down assays. In the immunoblot, 1% volumes of total cell lysate were run as input proteins to compare with proteins bound by the Vn96 peptides. The heat shock proteins tested are indicated. The right lower panel shows both HSP27 (indicated as ‘>’), HSP10 (indicated as ‘<<’) and a non-specific band (indicated as ‘*’).

Based on previous knowledge, we reasoned that an ideal HSP-binding peptide would be a 20–30 amino acid cationic peptide with hydrophobic side chains that favor strong interactions with the peptide binding cleft of Hsp70 isoforms [27], [28], [34], [35], [36] and other HSP paralogues. We designed a series of peptides that address the flexibility of basic and hydrophobic amino acids with sterically non-bulky residues. Among the candidate sequences screened, we identified peptides that yield complexes with HSPs from different organisms upon counter migration isoelectric focusing (IEF). Fine-tuning of the peptide sequences was carried out by synthesizing analogues of the most promising sequences, followed by their analysis using counter migration IEF. Here we show the results of these counter migration IEF experiments for two peptides (Vn20 and Vn96; depicted in Figure 1B). The peptides were placed at the anode of the IEF gel and recombinant HSPs placed at the cathode for counter migration. In the absence of counter migrating peptides, the recombinant HSP paralogues moved towards the anode of the IEF gel (Figure 1A). Upon counter migration with Vn peptides, recombinant HSPs were observed closer to the neutral spectrum of the pH gradient, representing complexes formed between the HSPs and the Vn peptides (Figure 1A). Unbound cationic Vn peptides migrated to the cathode end of the gel. A higher affinity of Vn96 over Vn20 for HSPs was observed as a higher proportion of HSP-Vn96 complexes formed compared to HSP-Vn20 complexes formed when similar quantities of both the peptides and the HSPs were loaded on the IEF. Based on these results, Vn96 was selected as a lead peptide for further experiments.

### The Vn96 peptide captures HSP complexes and enriches membrane-bound structures from total cell lysates

To further validate the specificity of the Vn96 peptide for HSPs, an affinity pull-down experiment equivalent to immunoprecipitation was designed using cell lysates from the breast cancer cell line MCF-7 prepared in the presence of 1% NP-40 detergent. Streptavidin-coupled Dynabeads were saturated with either biotinylated-Vn96 (b-Vn96) or a biotinylated scrambled sequence of Vn96 (the same amino acids, but arranged in a different order; b-Scr-Vn96), which were used to capture proteins from the cell lysate as described in the methods section. The bound complexes were washed extensively with cell lysis buffer and the captured proteins analyzed by immunoblotting for the indicated HSPs. As shown in Figure 1C, Vn96-coated beads were able to capture different members of the HSP family from the cell lysate, as indicated. In contrast, the scrambled sequence of Vn96 failed to capture the same HSP family members (Figure 1C). These results validated our design strategy and demonstrate that our Vn96 peptide specifically and efficiently binds to HSPs.

While performing the above-described pull-down experiments, we observed visible aggregation of b-Vn96 coated beads in the cell lysate. Both b-Vn96- and b-Scr-Vn96-coated magnetic beads had similar free-flowing suspension properties in lysis buffer, but this property changed for the b-Vn96 beads’ post-cell-lysate incubation. To investigate whether this aggregation was due to protein-protein interactions, aliquots of the samples were digested with Proteinase K (2 µg/ml final concentration). The Proteinase K digestion resulted in the beads becoming dispersed in suspension without any visible aggregation (data not shown). Because these aggregates were not observed in the b-Scr-Vn96-coated beads, this confirms Vn96-specific protein interactions. As HSPs are known to be associated with membrane domains on the surface, as well as inside cells [37], we analyzed the Proteinase K-digested supernatants by Transmission Electron Microscopy (TEM) for membrane structures. As shown in Text S1, the Proteinase K-digested supernatant from b-Vn96 samples showed a dense mass of vesicular structures, whereas no such structures were visible in the supernatants from the control sample (b-Scr-Vn96). These membrane structures resembled small vesicles of cytoplasmic origin [38], [39]. These data indicate that the Vn96 peptide can capture membrane-bound structures that are associated with HSPs.

### The Vn96 peptide precipitates HSP-associated membrane-bound structures from conditioned cell culture growth media

**Figure 2:**
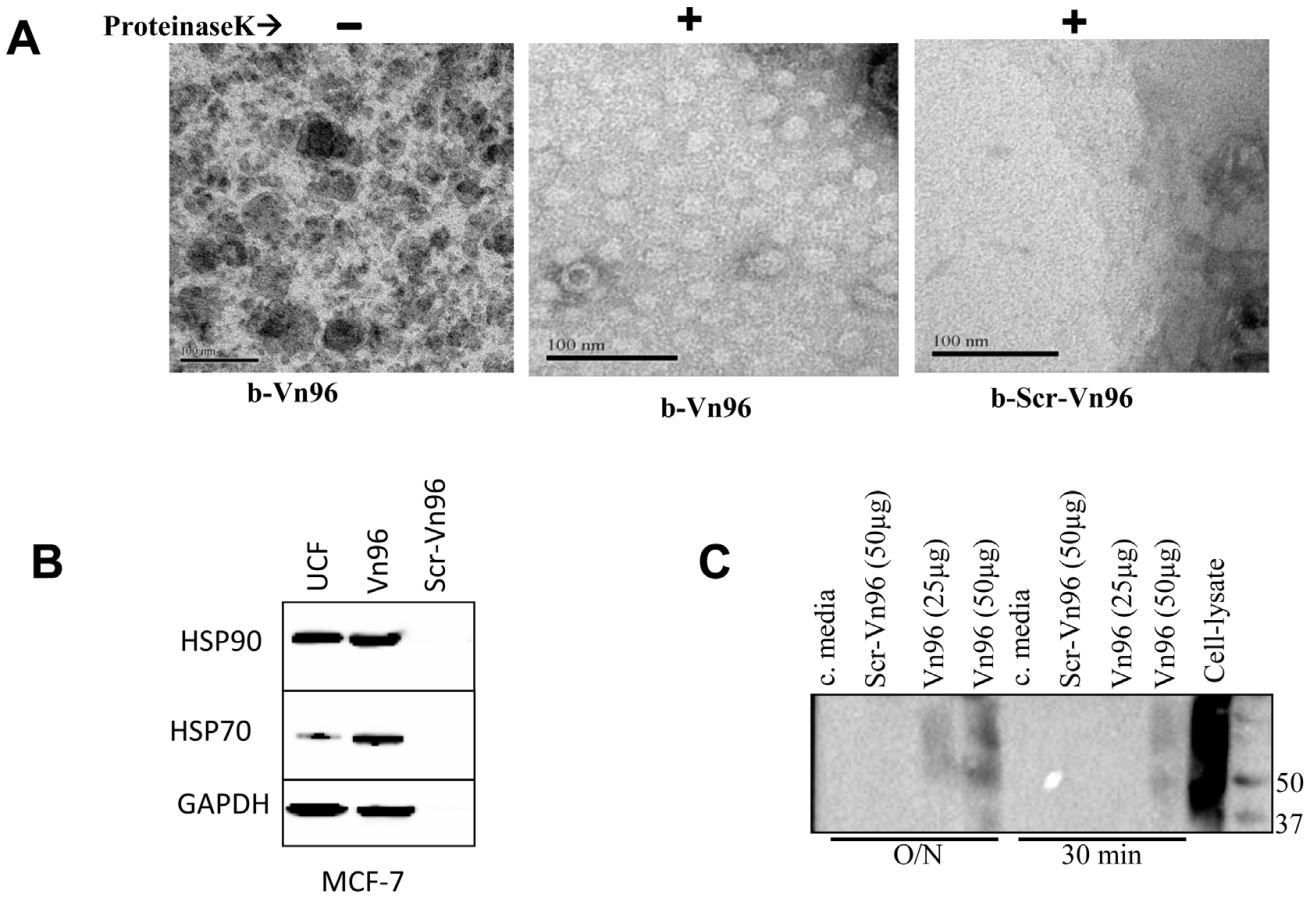
Characterization of extracellular materials precipitated by Vn96. A. Vn96 peptides precipitate vesicular structures from conditioned cell culture media. The biotinylated-Vn96 (b-Vn96) precipitated materials from conditioned cell culture media previously incubated with the MDA-MB-231 breast cancer cell line were subjected to Proteinase K digestion. The transmission electron microscopy analysis were performed on the precipitated material from the b-Vn96 sample (left panel), proteinase K-digested b-Vn96 sample (middle panel), and the proteinase K-digested sample from b-Scr-Vn96 (right panel). The scale bars are 100 nm. B. Identification of exosome markers in the Vn96-purified EVs from conditioned cell culture media. 50 µg each of Vn96 peptide and Scr-Vn96 were incubated with 1 ml of conditioned cell culture media previously incubated with the breast cancer cell line MCF-7 at 4°C for overnight. Exosomes were also isolated from the same conditioned cell culture media by ultracentrifugation (UCF). The presence of HSP70, HSP90 and GAPDH were assessed by immunoblotting. C. CD63 immunoblot. 1 ml of pre-cleared MCF-7 conditioned cell culture media was incubated to precipitate EVs with indicated amount of peptides either overnight (O/N) at 4°C or 30 minutes at room temperature. Total cell lysate of MCF-7 (equivalent to 0.2×10^6^ cells) was used as a positive control and conditioned cell culture media alone (c. media) was used as negative control. SDS-PAGE was performed in non-reducing conditions for CD63 immunoblots as recommended by the supplier.

Given the observation that Vn96 could capture HSPs that are associated with membranes, we chose to examine whether Vn96 could capture membrane-bound structures associated with extracellular HSPs from cell culture conditioned media. To generate conditioned media, the breast cancer cell line MDB-MB-231 was grown in EV-free standard cell culture media as described in the experimental procedures section and subsequently collected for downstream experiments. The conditioned growth media, as well as unused control growth media, was incubated overnight with rotation at 4°C with 100 µg/ml each of b-Vn96 or b-Scr-Vn96 peptide. Translucent precipitates were observed only in the b-Vn96 samples following centrifugation at 17,000×g. The 17,000×g pellets were washed with 1 ml of PBS three times (17,000×g) and the final pellets resuspended in 50 µl of PBS and treated with Proteinase K. TEM studies of Proteinase K-treated b-Vn96 samples revealed populations of vesicles, whereas the b-Scr-Vn96 samples showed no such structures (Figure 2A). The vesicular structures isolated using the b-Vn96 are reminiscent of previously described exosomes and EVs [40]. These results indicate the Vn96 peptide captures membrane-bound structures from cell culture growth media that are potentially EVs.

### Identification of canonical EV markers in Vn96-captured membrane structures from conditioned cell growth media

To determine if the membrane-bound structures isolated with b-Vn96 from conditioned cell culture media were indeed EVs, we examined material precipitated with the Vn96 peptide for protein markers of exosomes/EVs by Western-blot analysis. During the course of our experiments, we found that we could also precipitate membrane structures with the Vn96 peptide in the absence of biotinylation and linkage to magnetic beads, but using a similar centrifugation protocol (see methods); we therefore performed downstream experiments using this method.

Vn96 peptide or Scr-Vn96 peptide were added to pre-cleared conditioned cell culture growth media previously incubated with the breast cancer cell line MCF-7; materials were precipitated and harvested as described in the experimental procedures section. Exosomes were purified from the same conditioned cell culture growth media using ultracentrifugation (UCF) on a sucrose cushion as previously described [41]. Western-blot analysis of the material precipitated with Vn96 showed the presence of HSP70, HSP90, GAPDH (Figure 2B, lane 2), which were also present in the UCF-purified exosomes (Figure 2B, lane 1). Importantly, the amount of EV markers present in Vn96-precipitated material and UCF-purified material were comparable. No signal for EV markers was detected in material precipitated with the Vn96-Scr control peptide (Figure 2B, lane 3).

Similarly, the pre-cleared conditioned cell culture media from MCF-7 cells was incubated with the indicated amount of Vn peptides per ml either overnight (O/N) at 4°C or for 30 minutes at room temperature (Figure 2C). The precipitated materials were subjected to non-reducing SDS-PAGE, followed by anti-CD63 immunoblotting. Our results show that both the overnight and 30 minute incubation protocols precipitate EVs, but at different ratios of Vn96 peptide; specifically, less Vn96 peptide is required when the incubation time is prolonged at 4°C (Figure 2C). Together, these results show that we can precipitate EVs from cell culture growth media using the Vn96 peptide with efficiency comparable to UCF-mediated purification.

### The Vn96 peptide precipitates EVs from biological fluids

We wished to further explore whether Vn96 could capture EVs from sources other than cell culture growth media, such as biological fluids. We therefore chose to determine whether Vn96 could capture EVs from urine and plasma. Urine samples were collected from patients (consenting male patients scheduled for prostate biopsy) both pre- and post-digital rectal examination (DRE) with prostate massage. Plasma was collected from consenting healthy women and breast cancer patients.

**Figure 3:**
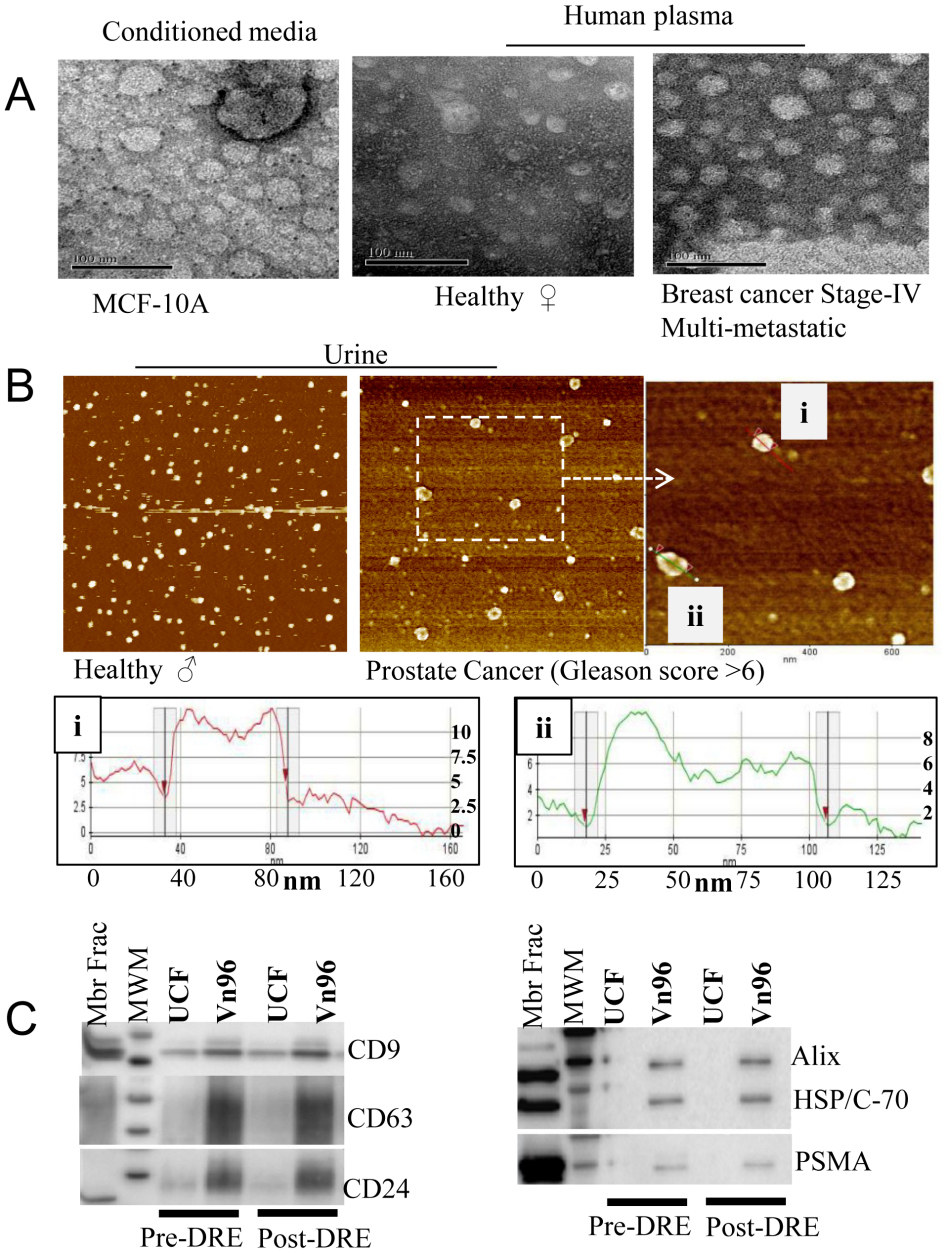
Visualization of Vn96 peptide-precipitated extracellular vesicles (EVs) from biological fluids. Pre-cleared biological fluids (conditioned cell culture media, human plasma and human urine) were used as described in experimental procedures. The Vn96 peptide-precipitated materials were dispersed into solution using Proteinase K digestion prior to microscopic analysis. A. Transmission electron microscopy images of Vn96-precipitated material from the indicated samples (conditioned cell culture media and diluted human plasma). The scale bars are 100 nm. B. Atomic force microscopy (phase) image of the Vn96 peptide-precipitated (Proteinase K digested) EVs from human urine. A differential size distribution pattern is observed between EVs from urine of normal and prostate cancer subjects (equal scale). The enlarged area from the prostate cancer image was used to measure width and thickness of two individual EVs (right panel, i and ii) in nanometers (nm) are shown in the bottom panel. C. Pre- and post-digital rectal exam with prostatic massage (DRE) urine samples were collected from consenting donors. EVs were isolated in parallel from equal volumes of urine using Vn96 and ultracentrifugation (UCF) methods; immunoblot analyses were performed using antibodies against the proteins indicated. Representative results for pre- and post-DRE urine samples from a donor are shown. Mbr Frac = Membrane fraction of prostate adenocarcinoma cell line LNCap and MWM = Protein molecular weight markers.

We first examined whether we could isolate membrane-bound structures from these materials with the Vn96 peptide using TEM and atomic force microscopy (AFM). The plasma samples were diluted ten-fold in PBS before being subjected to Vn96 peptide-mediated precipitation, whereas urine was left undiluted. All samples were subjected to pre-clearing by centrifugation at 17,000×g followed by filtration though 0.22 µm pore size filters. The pre-cleared samples were incubated with 50 µg/ml Vn96 or Scr-Vn96 peptide, followed by precipitation and washes with PBS as described in the methods section. The precipitates were subjected to Proteinase K digestion to obtain a homogenous dispersion of precipitated material, followed by TEM or AFM analyses. As shown in the TEM images (Figure 3A), the size distribution of the membrane structures was similar to the reported sizes of EVs (30 nm to 100 nm). Similarly, AFM analysis in tapping mode was performed for material precipitated from urine by Vn96 and the size distributions are shown in (Figure 3B). Nanoparticle tracking analysis (NTA) of all the samples prepared for Figure 3 was performed (Text S2). It is worth noting that the size distributions of the samples did not match with TEM or AFM measurements (Figures 3A and 3B and Text S2). This discrepancy may be due to the fact that NTA measures the sphere-equivalent hydrodynamic radius from scattered light, and biological samples such as EVs may have a significant hydration shell when dispersed in aqueous solutions, whereas TEM and AFM measure the dry physical structures only. Nonetheless, our results show that Vn96 is able to capture membrane-bound nanoparticles from biological fluids such as plasma and urine.

To determine if the material captured by Vn96 from biological fluids is indeed EVs, we performed Western-blot analysis for canonical EV protein markers. We first isolated material from equal volumes of urine in parallel using the Vn96 peptide and the UCF purification method and assessed for canonical protein markers of EVs by Western-blot analysis (Figure 3C). Urinary EVs precipitated with Vn96 contained canonical EV protein markers (CD9, CD63, CD24, Hsp70, Alix) of comparable or greater abundance than the corresponding UCF-purified exosome sample. Prostate-specific marker, FOLH1 (PSMA) was also detected in post-DRE Vn96-precipitated EVs from the urine of prostate cancer subjects. These data indicate that the Vn96 peptide can precipitate EVs from biological fluids, such as urine.

### Comparative proteomic profiling of Vn96-captured EVs from conditioned cell culture growth media and human plasma

To determine if Vn96-mediated capture of EVs results in the isolation of a similar population of EVs as UCF-mediated exosome purification we performed comparative proteomic profiling studies on material isolated from conditioned cell culture growth media and plasma using these methods. For the comparative proteomic studies we used conditioned cell culture growth media used to propagate MCF-10A, MCF-7 and MDA-MB-231 mammary cell lines, which were divided into aliquots that were subjected to each preparation method.

EVs and exosomes were harvested using Vn96 or UCF as described in previous sections. The collected EVs were processed as described in the experimental procedures section. Q-Exactive quadrupole-orbitrap mass spectrometer (Thermo-Fisher Scientific, San Jose, CA) generated spectra were used to search a UniProt protein database with the SEQUEST algorithm (Proteome Discoverer 1.3). Search results were further submitted to Scaffold 4 (Proteome Software, Portland, OR) to generate a minimal list of non-redundant proteins. We extracted the proteome from each sample with 100% probable candidates for Gene Ontology (GO) analysis. As shown in Table 1, GO analysis for cellular components with the proteomes from each sample showed that they originate from extracellular membrane-bound vesicles. More importantly, the proteomes of Vn96-extracted EVs from conditioned cell culture growth media and plasma samples showed highly significant *p*-values for both the GO terms ‘extracellular vesicular exosome’ (GO:0070062) and ‘Extracellular membrane-bounded organelle’ (GO:0065010). Moreover, the proteomes of EVs captured with Vn96 showed a good comparison to the UCF-purified exosome proteomes from the conditioned cell culture growth media or plasma samples as shown in Table 1. These results demonstrate that Vn96 captures a population of EVs that are very similar to exosomes that are purified using the classical UCF method.

**Table 1:** Gene list enrichment analysis for Cellular Component Ontology using ToppGene.

GO:0070062: Extracellular vesicular Exosome			
Sample	EV isolation	% GO term	p-value
Conditioned media: MCF-10A	Vn96	41.27	1.83E-30
Conditioned media: MCF-10A	UC	7.94	4.07E-06
Conditioned media: MCF-7	Vn96	23.81	6.07E-11
Conditioned media: MCF-7	UC	7.94	1.20E-04
Conditioned media: MDA-MB-231	Vn96	20.63	6.53E-19
Conditioned media: MDA-MB-231	UC	20.36	4.07E-19
Human plasma-1	Vn96	14.29	1.99E-10
Human plasma-2	Vn96	12.7	2.59E-08
Human plasma-3	Vn96	15.87	7.92E-11
Human plasma-4	Vn96	11.11	6.70E-19
**GO:0065010: Extracellular membrane-bounded organelle**			
Conditioned media: MCF-10A	Vn96	41	5.03E-30
Conditioned media: MCF-10A	UC	7.94	5.61E-08
Conditioned media: MCF-7	Vn96	23.08	9.98E-11
Conditioned media: MCF-7	UC	7.69	1.41E-04
Conditioned media: MDA-MB-231	Vn96	20.63	6.53E-19
Conditioned media: MDA-MB-231	UC	20.36	4.07E-19
Human plasma-1	Vn96	13.85	2.68E-10
Human plasma-2	Vn96	12.31	3.36E-08
Human plasma-3	Vn96	15.38	1.10E-10
Human plasma-4	Vn96	10.77	8.38E-07

The list of 100% probable proteins from each sample’s proteome was derived and gene list enrichment analysis was carried out using ToppFun (https://toppgene.cchmc.org/) for Cellular Component ontology. ToppGene Suite is being developed at Division of Biomedical Informatics, Cincinnati Children's Hospital Medical Center (BMI CCHMC), Cincinnati, OH 45229. For comparison we also analysed results from two proteomic data-sets [Vesiclepedia ID_44 and Vesiclepedia ID_353] derived from exosomes purified from human plasma using Size exclusion filtration followed by Sucrose density gradient ultracentrifugation (UC), as posted on Vesiclepedia (http://microvesicles.org/index.html). Cellular component ontology analysis using ToppFun (GO:0070062: Extracellular vesicular Exosome) for Vesiclepedia ID_44 and Vesiclepedia ID_353 derived exosomal proteome revealed p-values of 1.15E-09 and 1.92E-11 respectively. Similar analysis for GO:0065010 (Extracellular membrane-bounded organelle) from Vesiclepedia ID_44 and Vesiclepedia ID_353 derived exosomal proteome revealed p-values of 1.54E-09 and 2.66E-11 respectively. The %GO term means the percentage ratio of ‘list of proteins as input’ over the assigned list of genes for a specific annotation.

### Comparative miRNA and other long RNA profiling of Vn96-captured EVs from conditioned cell culture growth media

We wished to further validate that Vn96 isolates a similar population of EVs as other methods. Therefore, we chose to compare the miRNA as well as total RNA cargo of EVs/exosomes purified by different methods (Vn96, UCF, and a commercially-available reagent) from conditioned cell culture growth media used to propagate two breast cancer cell lines, MCF-7 and MDA-MB-231. RNA libraries prepared from isolated EVs were sequenced on the Ion Proton platform (Life Technologies) according to the manufacturer’s recommendations, with slight modifications as described in the methods section. Normalization of long RNAs and small RNAs was performed using Reads per Kilobase per Million mapped reads (RPKM) and Trimmed Mean of M-values (TMM) or Lowess methods, respectively. The steps followed for data processing and analysis for profiling the expression of all RNAs and microRNAs is presented as a flowchart in Text S3. The RNA sequence data is archived at Gene Expression Omnibus data repository [GSE58464].

**Figure 4:**
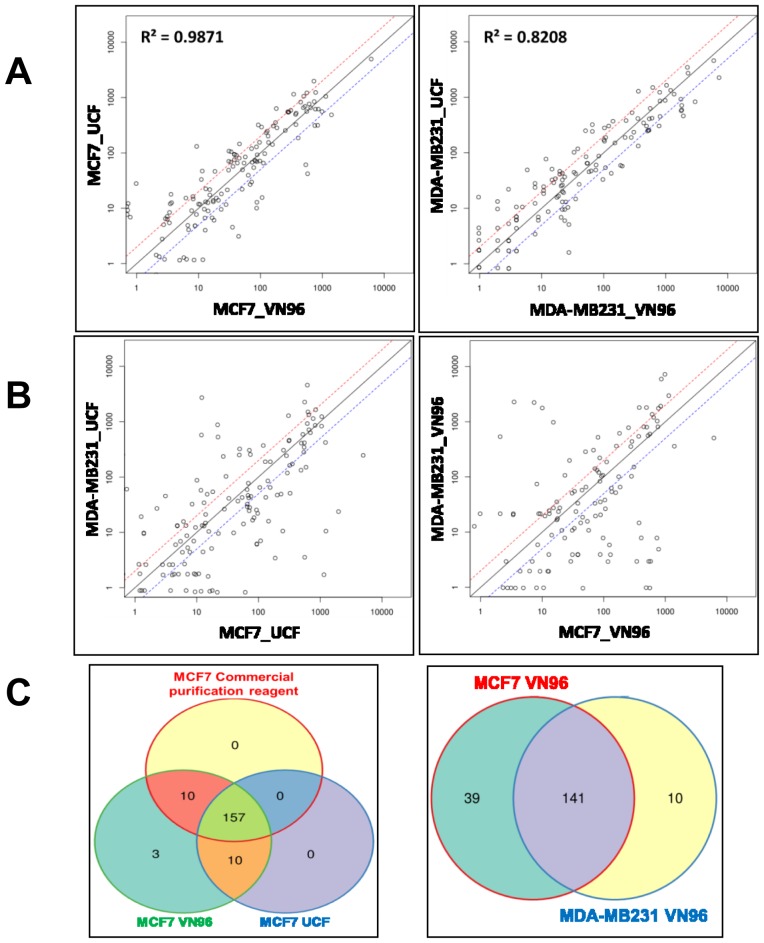
Comparative miRNA-seq data for Vn96- and UCF-purified EVs from conditioned cell culture media. A. Scatter plot comparing normalized expression profiles of miRNAs contained in EVs isolated from the indicated conditioned cell culture media using either ultracentrifugation or the Vn96 peptide. For example, MCF7_UCF and MCF7_VN96 indicate that EVs were purified from conditioned cell culture media previously incubated with MCF-7 cells by ultracentrifugation and the Vn96 peptide, respectively. High Pearson correlations between ultracentrifugation and Vn96 peptide methods of EV purification from the same sample validate Vn96 as an EV purification tool. B. Scatter plot comparing normalized expression profiles of miRNAs contained in EVs isolated from MCF-7 versus MDA-MB-231 conditioned cell culture media using the same purification method. C. Venn diagram of miRNAs contained in EVs isolated from MCF-7 conditioned cell culture media using different methods (ultracentrifugation, Vn96 peptide and a commercially-available exosome purification kit). Less than 10% differences were observed in the miRNA populations between the ultracentrifugation and Vn96 peptide methods, and the commercial kit and Vn96 peptide methods (left panel), but a wider variation in miRNA populations was observed in EVs from different cell lines (right panel).

Comparative assessments of miRNA extracted from EVs isolated using Vn96 and the UCF method for one cell line-type revealed very similar profiles with high Pearson correlations, minimal expression variation and less than 5% population variability (Figure 4A). On the other hand, higher dispersion, differential expression and high population variability were observed when miRNA cargos of UCF-purified EVs were profiled for two different cell lines (Figure 4B). Similar wide variations were also observed in the miRNA profiles of EVs precipitated from the same two cell lines using the Vn96 peptide. Furthermore, populations of differentially-expressed miRNAs identified in the EVs of the two cell lines were highly similar irrespective of the isolation method used (UCF or Vn96) as shown in the normalized heat map in Text S4.

Identified miRNAs extracted from MCF-7 cell-line EVs obtained using different methods of EV isolation (including a commercially available EV isolation kit) showed minimal diversifications, as shown in the Venn diagram in Figure 4C (left panel). Greater diversification was observed when the populations of identified miRNA cargos were compared between different cell lines (Figure 4C right panel and Text S5). The highly similar miRNA profiles observed between Vn96 and UCF methods of EV purification from conditioned cell culture growth media further validate Vn96 as a highly specific tool to enrich EVs. RNA profiles of EVs typically show a characteristic enrichment of different species of RNAs (miRNA, miscRNA and lincRNA etc) [42] that differ from total cellular RNA species profiles. For example, the proportion of rRNA is usually decreased by several-fold in EVs in comparison to its proportion in total cellular RNA. Our RNA sequence data reveal similar characteristic patterns [42] of different species of RNAs when compared to UCF and Vn96 methods of EV purification (Text S6).

Together, our data show that Vn96 captures EVs that contain a RNA cargo content that is similar to the established UCF purification method and a commercially-available EV isolation kit.

## Discussion

We initially set out to develop HSP-binding peptides that could be used to capture extracellular HSP complexes for further investigation. Our observations during the validation of the peptides led us to discover their potential as exosome or EV capture tools. We found that the Vn96 peptide could capture EVs from conditioned cell culture growth media and biological fluids, such as urine and plasma. Our recent unpublished results also show that Vn96 can capture EVs from mouse and canine plasma, as well as from bovine milk (data not shown). Importantly, we demonstrate that Vn96-mediated EV capture permits the collection of EVs that are both physically and cargo-content similar to EVs/exosomes isolated by the standard UCF-purification method and a commercially-available EV isolation kit. Unlike other methods, Vn96 permits the collection of EVs from multiple fluid sources using standard laboratory equipment in a minimal amount of time (<40 minutes).

While characterizing Vn96’s ability to capture extracellular HSP complexes we observed visibly distinct aggregation patterns in conditioned cell culture growth media and biological fluids when Vn96 was added. We observed no visible aggregation in stock solutions of the peptides (Vn96 and Scrambled-Vn96 in PBS) or the samples to which Scrambled-Vn96 was added. This observation prompted us to investigate the constituents and nature of the aggregates induced by the Vn96 peptide in pre-cleared conditioned cell culture growth media, urine and plasma. We found that Vn96 acts like a ‘nano-probe’, which enriches vesicular structures that have the properties of exosomes and/or microvesicles (collectively, EVs). We compared Vn96-captured material to exosomes purified by ultracentrifugation using NTA, TEM, AFM, immunoblotting, next-generation sequencing of miRNA cargo, and proteome-based cellular component ontology analysis, and found that they are indeed EVs. Moreover, because the Vn96 peptide can bind to HSPs from multiple species (see Figure 1A), its ability to capture EVs may not be limited to human biological fluids and cell culture samples. Vn96-mediated EV capture may therefore be applicable to basic research using animal models, as well as diagnostic methods for animal health.

We believe that Vn96 is able to capture EVs due to its interaction with HSPs on their surface, since EV-mediated extracellular transport of HSPs occurs in many pathological conditions [5]–[15]. However, by virtue of its design the Vn96 peptide forms a cationic alpha helix at physiologically relevant salt and buffer conditions, which may allow Vn96 to gain overall avidity towards ultra-small subcellular structures and other molecules from intracellular and extracellular origin. It is known that alpha-helical cationic peptides can aggregate small multilayered lipid vesicles based on the peptide’s ability to form a helical coiled-coil [43] that interacts with and/or inserts into membranes [44]–[46]; therefore, we cannot rule out the possibility that the cationic nature of the Vn96 peptide may allow it to directly interact with the membranes of EVs to facilitate their capture. Nonetheless, all of our results confirm that the Vn96 peptide is a useful tool for the collection of EVs from wide variety of sample types, and captures EVs that have characteristics that are equivalent to those obtained by the standard ultracentrifugation isolation method.

The release of EVs is a conserved and essential process of diverse prokaryotic and eukaryotic cells. But this essential process is co-opted during cancer, in which EVs play critical roles in the establishment of cell transformation, cancer progression, metastasis, distal niche formation, stemness, and many aspects of tumor cross-talk with surrounding cells [47]. There is ample evidence that cancer cells produce EVs with cancer-specific signatures, which can be found in body fluids, a finding that opens up new frontiers for cancer diagnostics research. A method that allows the simple and rapid capture of EVs, such as the Vn96 peptide, will permit significant advancement of this field. However, the release of EVs that contain disease signatures is not limited to cancer. Neurons with infectious prion proteins were found to produce EVs that contain the same prions [48]. Similarly, virally-infected host cells release EVs that contain viral factors [49]–[53], which influence host response. Therefore, the capture of EVs from body fluids represents a possible new approach to minimally-invasive broad-based disease diagnostics.

Vn96-based EV purification provides a simple, efficient, and rapid method of EV enrichment and capture. There are potential benefits of EV enrichment with the Vn96 peptide for both established diagnostics and for new biomarker discovery. Current obstacles to the application of EVs in the clinical setting include difficulties with isolation methods and most prominently enrichment of disease-specific EVs from complex mixtures of vesicular material originating from various cell/tissue types. The current methods available for the isolation of EVs are based on physical characteristics, which can be efficient but are time-consuming, require specialized equipment, and may lack specificity. Similarly, affinity-based methods such as the use of antibody capture are still based on EV ‘markers’, which appear to vary amongst EV populations [54] and may therefore not be present on all EV species. We have demonstrated that the Vn96 peptide isolates EVs that have clinical value and that the Vn96 peptide compares favourably to current isolation methods in terms of efficiency, cost, and platform versatility as an EV capture tool for discovery research, animal health, and clinical applications.

## Supporting Information

Text S1 **The Vn96 peptide enriches membrane-bound structures from total cell lysates.** A portion of pull-down material shown in Figure 1B was washed with PBS and subjected to Proteinase K digestion. The beads were removed and the suspension was subjected to transmission electron microscopy analysis. A dense vesicular aggregated material, resembling different subcellular vesicles, was observed in the samples from the b-Vn96 pull-down. No such structures were observed in b-Scr-Vn96 samples. The scale bars are 100 nm.
(PDF)

Text S2 **Particle size distribution: nanoparticle tracking analysis.** The size distribution and relative abundances of the EVs from the samples shown in Figure 3 were measured using nanoparticle tracking analysis as described in the experimental procedures.
(PDF)

Text S3 **Flowchart for the analysis of the next-generation RNA-sequencing.** Flowchart for the analysis of the next-generation sequencing data for profiling RNA and microRNA expression. RNA libraries prepared from EVs isolated with different methods were sequenced on the Proton platform (Life Technologies). Normalization of long RNA was realized with Reads Per Kilobase per Million mapped reads (RPKM) and small-RNA were normalized with Trimmed Mean of M-values (TMM) or Lowess methods.
(PDF)

Text S4 **Heatmap showing the abundance of miRNA.** Heatmap showing the abundance of miRNA contained in EVs produced by MCF-7 and MDA-MB-231 cell lines (abundance values normalized with Lowess method). Different methods to isolate EVs were compared (Ultra for ultracentrifugation, VN96 for Vn peptide method, and C.K for commercially-available kit). Missing values are indicated by the grey color. Only miRNAs with zero reads are treated as missing values, whereas miRNAs with 1 or 2 reads are shown in the heatmap.
(PDF)

Text S5 **Venn diagram of comparative miRNA expression.** miRNA-seq on EVs isolated ultracentrifugation or the Vn96 peptide from cell culture media previously incubated with two different breast cancer cell lines (MCF-7 and MDA-MB-231). Venn diagram comparing miRNA expression between EVs isolated from MCF7 and MDA-MB-231, and between ultracentrifugation and Vn96 peptide methods.
(PDF)

Text S6 **Comparative distribution of RNA species contained in EVs.** RNA species contained in EVs produced by breast cancer cell lines MCF-7 and MDA-MB-231 that were isolated by ultracentrifugation or the Vn96 peptide method. The right figure is an enlargement of the left figure in order to facilitate the visualization of less abundant RNA species. Proportions of RNA species are similar between isolation methods used. We also observed an enrichment of some RNA species in EVs compared to RNA species contained in the cell. (rRNA represent around 1–10% of all RNA in EVs, while in a cell more than 90% of RNA are rRNA).
(PDF)
