# Supplementary material for: Rapid Isolation of Extracellular Vesicles from Cell Culture and Biological Fluids Using a Synthetic Peptide with Specific Affinity for Heat Shock Proteins
Source: PLoS One. 2014 Oct 17;9(10):e110443. doi: 10.1371/journal.pone.0110443 (PMC4201556; doi:10.1371/journal.pone.0110443)
Supplement: Text S1 — The Vn96 peptide enriches membrane-bound structures from total cell lysates. A portion of pull-down material shown in Figure 1B was washed with PBS and subjected to Proteinase K digestion. The beads were removed and the suspension was subjected to transmission electron microscopy analysis. A dense vesicular aggregated material, resembling different subcellular vesicles, was observed in the samples from the b-Vn96 pull-down. No such structures were observed in b-Scr-Vn96 samples. The scale bars are 100 nm. (PDF) [file pone.0110443.s001.pdf]

Supporting information: S1

The Vn96 peptide enriches membrane-bound structures from total cell lysates

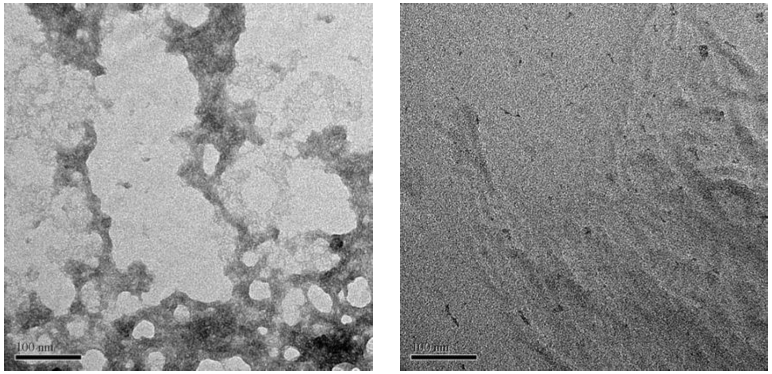

b-Vn96 **b-Scr-Vn96**

A portion of pull-down material shown in Figure 1B was washed with PBS and subjected to Proteinase K digestion. The beads were removed and the suspension was subjected to transmission electron microscopy analysis. A dense vesicular aggregated material, resembling different subcellular vesicles, was observed in the samples from the b-Vn96 pull-down. No such structures were observed in b-Scr-Vn96 samples. The scale bars are 100nm.
