# Supplementary material for: Rapid Isolation of Extracellular Vesicles from Cell Culture and Biological Fluids Using a Synthetic Peptide with Specific Affinity for Heat Shock Proteins
Source: PLoS One. 2014 Oct 17;9(10):e110443. doi: 10.1371/journal.pone.0110443 (PMC4201556; doi:10.1371/journal.pone.0110443)
Supplement: Text S2 — Particle size distribution: nanoparticle tracking analysis. The size distribution and relative abundances of the EVs from the samples shown in Figure 3 were measured using nanoparticle tracking analysis as described in the experimental procedures. (PDF) [file pone.0110443.s002.pdf]

Supporting information: S2

Particle size distribution : nanoparticle tracking analysis

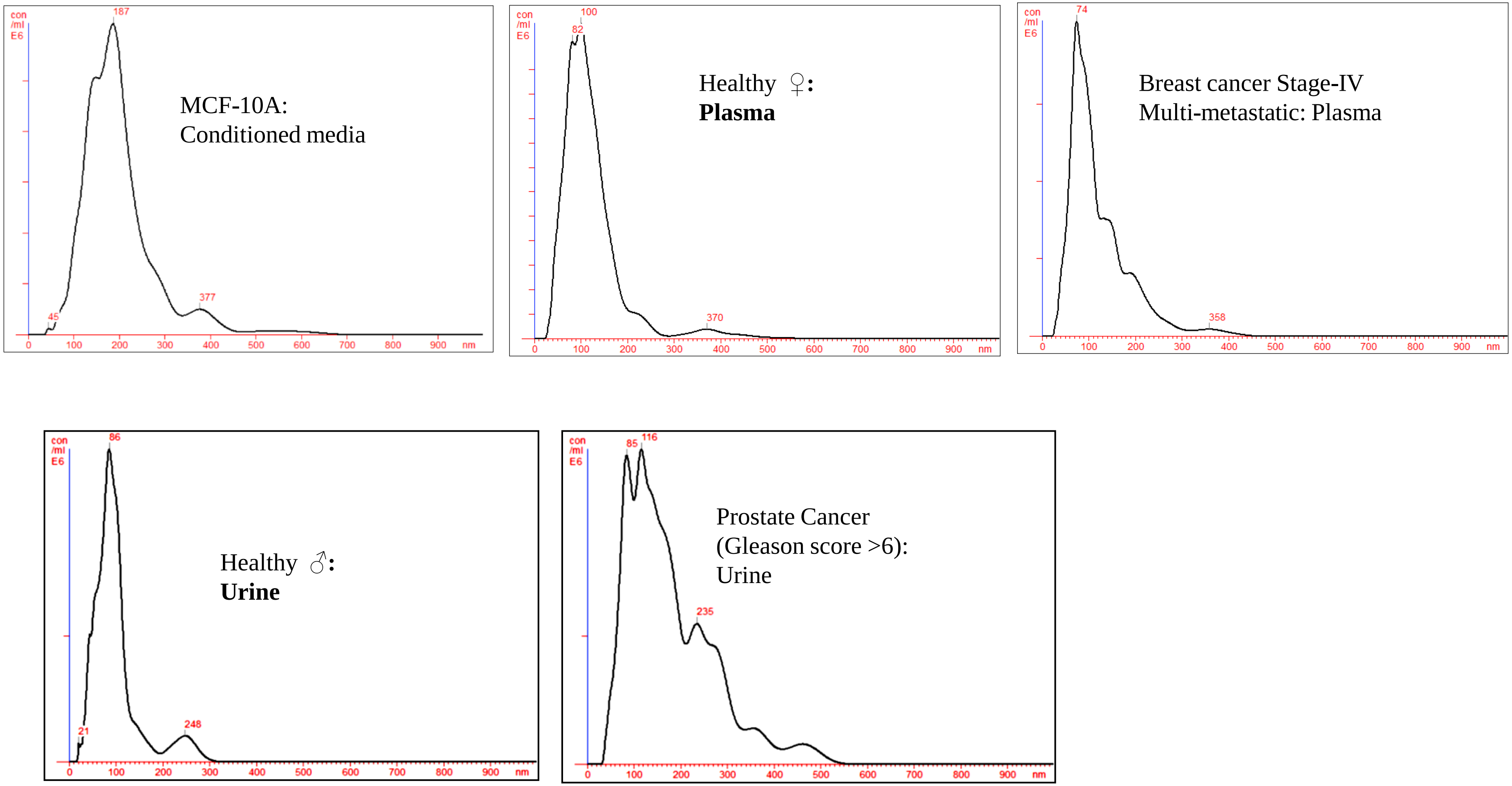

The size distribution and relative abundances of the EVs from the samples shown in Figure 3 were measured using nanoparticle tracking analysis as described in the experimental procedures.
