# Supplementary material for: Rapid Isolation of Extracellular Vesicles from Cell Culture and Biological Fluids Using a Synthetic Peptide with Specific Affinity for Heat Shock Proteins
Source: PLoS One. 2014 Oct 17;9(10):e110443. doi: 10.1371/journal.pone.0110443 (PMC4201556; doi:10.1371/journal.pone.0110443)
Supplement: Text S3 — Flowchart for the analysis of the next-generation RNA-sequencing. Flowchart for the analysis of the next-generation sequencing data for profiling RNA and microRNA expression. RNA libraries prepared from EVs isolated with different methods were sequenced on the Proton platform (Life Technologies). Normalization of long RNA was realized with Reads Per Kilobase per Million mapped reads (RPKM) and small-RNA were normalized with Trimmed Mean of M-values (TMM) or Lowess methods. (PDF) [file pone.0110443.s003.pdf]

Supporting information: S3

Flowchart for the analysis of the next-generation RNA-sequencing

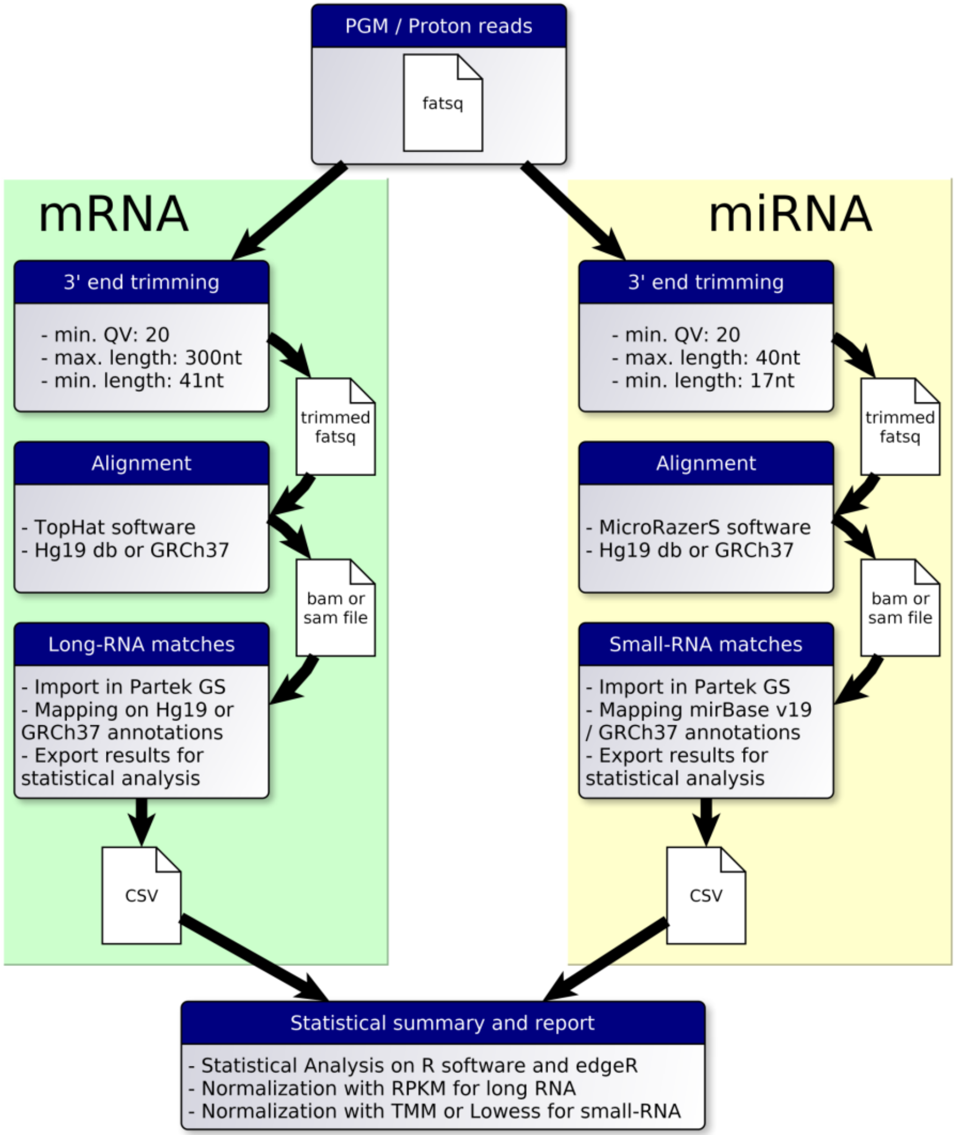

Flowchart for the analysis of the next-generation sequencing data for profiling RNA and microRNA expression. RNA libraries prepared from EVs isolated with different methods were sequenced on the Proton platform (Life Technologies). Normalization of long RNA was realized with Reads Per Kilobase per Million mapped reads (RPKM) and small-RNA were normalized with Trimmed Mean of M-values (TMM) or Lowess methods.
