# Supplementary material for: Rapid Isolation of Extracellular Vesicles from Cell Culture and Biological Fluids Using a Synthetic Peptide with Specific Affinity for Heat Shock Proteins
Source: PLoS One. 2014 Oct 17;9(10):e110443. doi: 10.1371/journal.pone.0110443 (PMC4201556; doi:10.1371/journal.pone.0110443)
Supplement: Text S4 — Heatmap showing the abundance of miRNA. Heatmap showing the abundance of miRNA contained in EVs produced by MCF-7 and MDA-MB-231 cell lines (abundance values normalized with Lowess method). Different methods to isolate EVs were compared (Ultra for ultracentrifugation, VN96 for Vn peptide method, and C.K for commercially-available kit). Missing values are indicated by the grey color. Only miRNAs with zero reads are treated as missing values, whereas miRNAs with 1 or 2 reads are shown in the heatmap. (PDF) [file pone.0110443.s004.pdf]

Supporting information: S4

Heatmap showing the abundance of miRNA

Heatmap showing the abundance of miRNA contained in EVs produced by MCF-7 and MDA-MB-231 cell lines (abundance values normalized with Lowess method). Different methods to isolate EVs were compared (Ultra for ultracentrifugation, VN96 for Vn peptide method, and C.K for commercially-available kit). Missing values are indicated by the grey color. Only miRNAs with zero reads are treated as missing values, whereas miRNAs with 1 or 2 reads are shown in the heatmap.

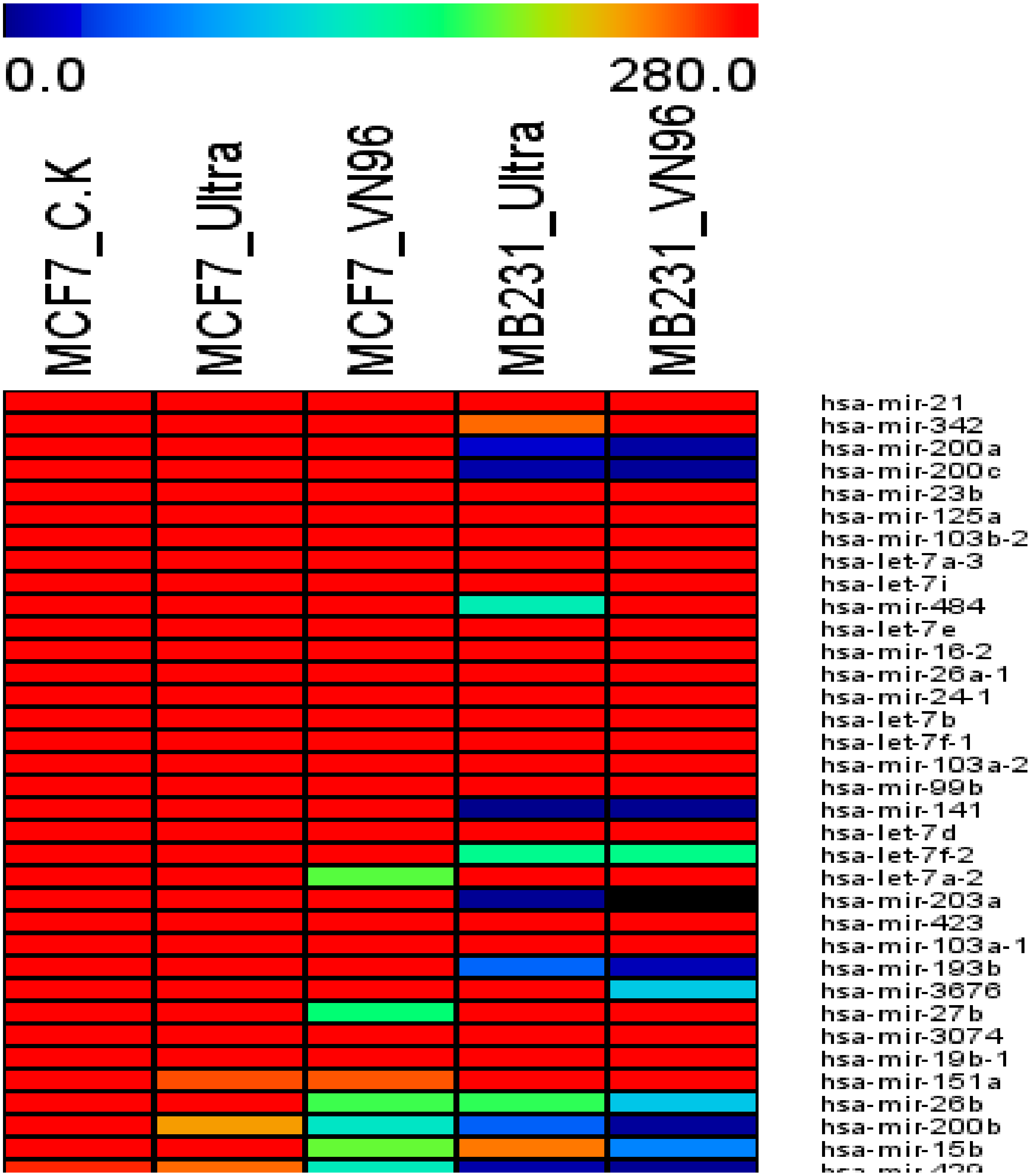

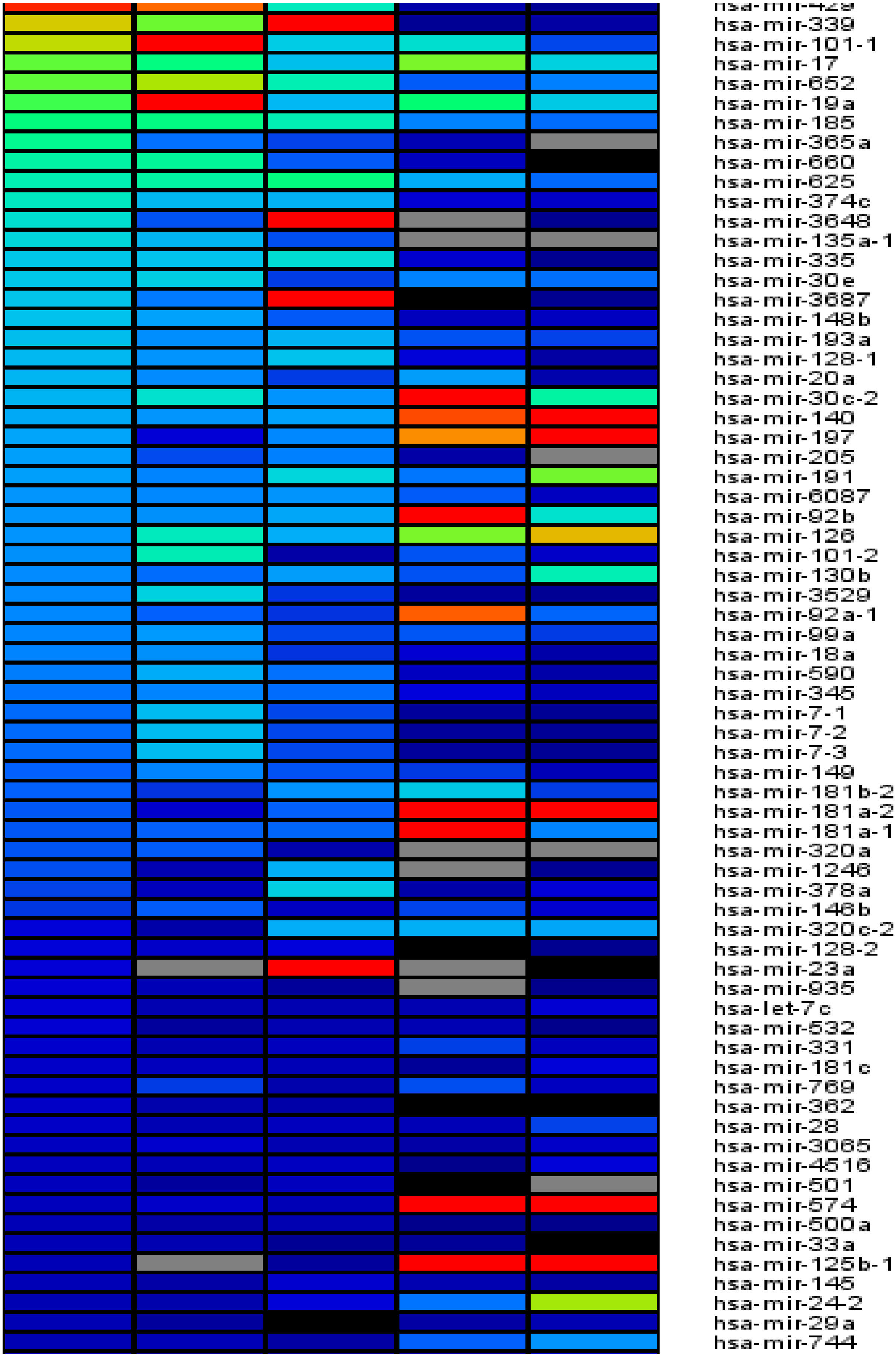
hsa-mir-339
hsa-mir-101-1
hsa-mir-17
hsa-mir-652
hsa-mir-19a
hsa-mir-185
hsa-mir-365a
hsa-mir-660
hsa-mir-625
hsa-mir-374c
hsa-mir-3648
hsa-mir-135a-1
hsa-mir-335
hsa-mir-30e
hsa-mir-3687
hsa-mir-148b
hsa-mir-193a
hsa-mir-128-1
hsa-mir-20a
hsa-mir-30c-2
hsa-mir-140
hsa-mir-197
hsa-mir-205
hsa-mir-191
hsa-mir-6087
hsa-mir-92b
hsa-mir-126
hsa-mir-101-2
hsa-mir-130b
hsa-mir-3529
hsa-mir-92a-1
hsa-mir-99a
hsa-mir-18a
hsa-mir-590
hsa-mir-345
hsa-mir-7-1
hsa-mir-7-2
hsa-mir-7-3
hsa-mir-149
hsa-mir-181b-2
hsa-mir-181a-2
hsa-mir-181a-1
hsa-mir-320a
hsa-mir-1246
hsa-mir-378a
hsa-mir-146b
hsa-mir-320c-2
hsa-mir-128-2
hsa-mir-23a
hsa-mir-935
hsa-let-7c
hsa-mir-532
hsa-mir-331
hsa-mir-181c
hsa-mir-769
hsa-mir-362
hsa-mir-28
hsa-mir-3065
hsa-mir-4516
hsa-mir-501
hsa-mir-574
hsa-mir-500a
hsa-mir-33a
hsa-mir-125b-1
hsa-mir-145
hsa-mir-24-2
hsa-mir-29a
hsa-mir-744

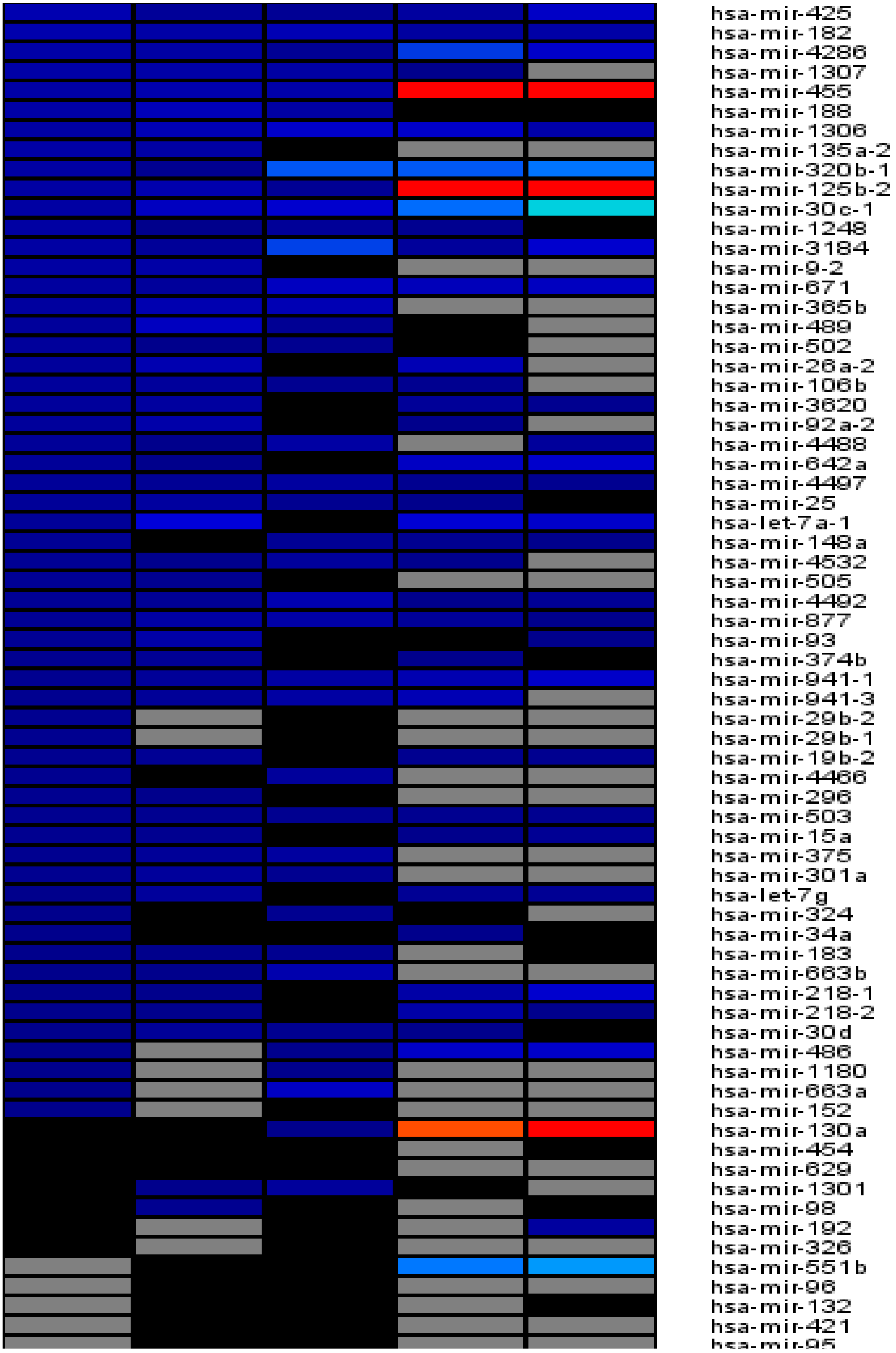
hsa-mir-425
hsa-mir-182
hsa-mir-4286
hsa-mir-1307
hsa-mir-455
hsa-mir-188
hsa-mir-1306
hsa-mir-135a-2
hsa-mir-320b-1
hsa-mir-125b-2
hsa-mir-30c-1
hsa-mir-1248
hsa-mir-3184
hsa-mir-9-2
hsa-mir-671
hsa-mir-365b
hsa-mir-489
hsa-mir-502
hsa-mir-26a-2
hsa-mir-106b
hsa-mir-3620
hsa-mir-92a-2
hsa-mir-4488
hsa-mir-642a
hsa-mir-4497
hsa-mir-25
hsa-let-7a-1
hsa-mir-148a
hsa-mir-4532
hsa-mir-505
hsa-mir-4492
hsa-mir-877
hsa-mir-93
hsa-mir-374b
hsa-mir-941-1
hsa-mir-941-3
hsa-mir-29b-2
hsa-mir-29b-1
hsa-mir-19b-2
hsa-mir-4466
hsa-mir-296
hsa-mir-503
hsa-mir-15a
hsa-mir-375
hsa-mir-301a
hsa-let-7g
hsa-mir-324
hsa-mir-34a
hsa-mir-183
hsa-mir-663b
hsa-mir-218-1
hsa-mir-218-2
hsa-mir-30d
hsa-mir-486
hsa-mir-1180
hsa-mir-663a
hsa-mir-152
hsa-mir-130a
hsa-mir-454
hsa-mir-629
hsa-mir-1301
hsa-mir-98
hsa-mir-192
hsa-mir-326
hsa-mir-551b
hsa-mir-96
hsa-mir-132
hsa-mir-421

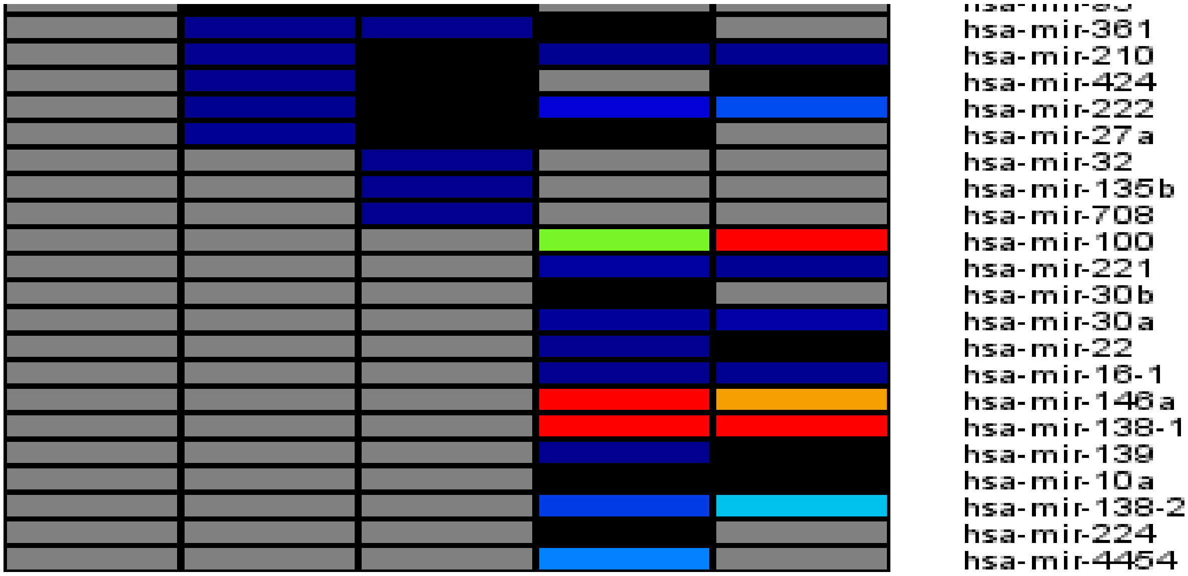
hsa-mir-361
hsa-mir-210
hsa-mir-424
hsa-mir-222
hsa-mir-27a
hsa-mir-32
hsa-mir-135b
hsa-mir-708
hsa-mir-100
hsa-mir-221
hsa-mir-30b
hsa-mir-30a
hsa-mir-22
hsa-mir-16-1
hsa-mir-146a
hsa-mir-138-1
hsa-mir-139
hsa-mir-10a
hsa-mir-138-2
hsa-mir-224
hsa-mir-4454
