# Supplementary material for: Rapid Isolation of Extracellular Vesicles from Cell Culture and Biological Fluids Using a Synthetic Peptide with Specific Affinity for Heat Shock Proteins
Source: PLoS One. 2014 Oct 17;9(10):e110443. doi: 10.1371/journal.pone.0110443 (PMC4201556; doi:10.1371/journal.pone.0110443)
Supplement: Text S5 — Venn diagram of comparative miRNA expression. miRNA-seq on EVs isolated ultracentrifugation or the Vn96 peptide from cell culture media previously incubated with two different breast cancer cell lines (MCF-7 and MDA-MB-231). Venn diagram comparing miRNA expression between EVs isolated from MCF7 and MDA-MB-231, and between ultracentrifugation and Vn96 peptide methods. (PDF) [file pone.0110443.s005.pdf]

**Supporting information: S5**

## Venn diagram of comparative miRNA expression

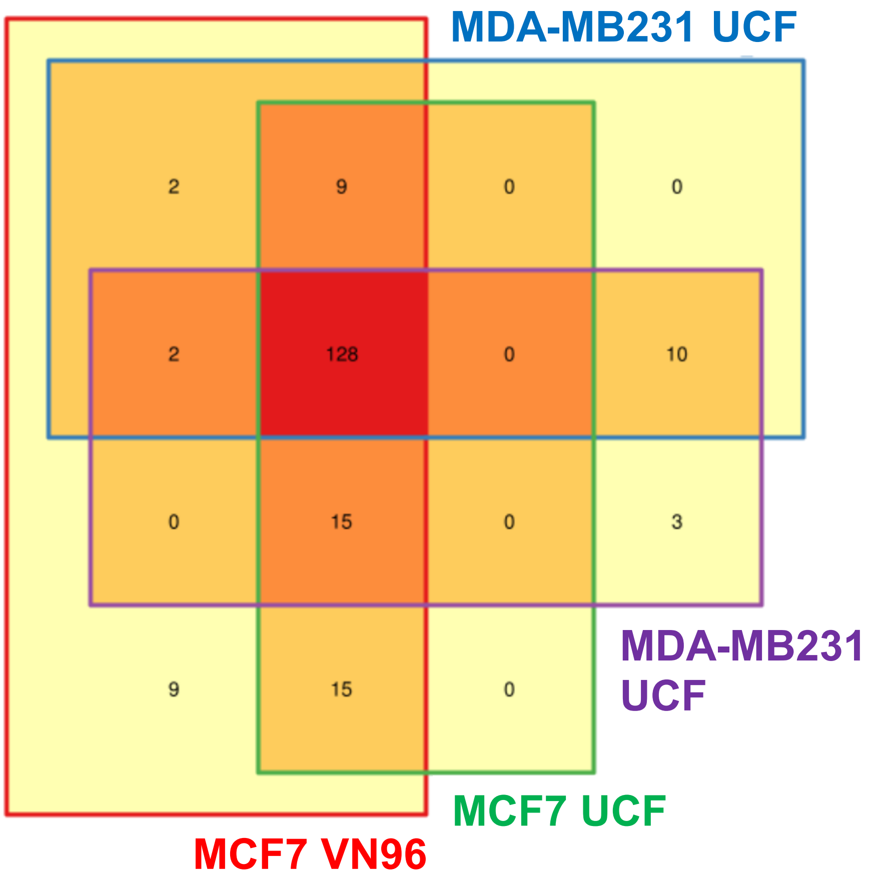


miRNA-seq on EVs isolated ultracentrifugation or the Vn96 peptide from cell culture media previously incubated with two different breast cancer cell lines (MCF-7 and MDA-MB-231). Venn diagram comparing miRNA expression between EVs isolated from MCF7 and MDA-MB-231, and between ultracentrifugation (UCF) and Vn96 peptide methods.
