# Supplementary material for: Rapid Isolation of Extracellular Vesicles from Cell Culture and Biological Fluids Using a Synthetic Peptide with Specific Affinity for Heat Shock Proteins
Source: PLoS One. 2014 Oct 17;9(10):e110443. doi: 10.1371/journal.pone.0110443 (PMC4201556; doi:10.1371/journal.pone.0110443)
Supplement: Text S6 — Comparative distribution of RNA species contained in EVs. RNA species contained in EVs produced by breast cancer cell lines MCF-7 and MDA-MB-231 that were isolated by ultracentrifugation or the Vn96 peptide method. The right figure is an enlargement of the left figure in order to facilitate the visualization of less abundant RNA species. Proportions of RNA species are similar between isolation methods used. We also observed an enrichment of some RNA species in EVs compared to RNA species contained in the cell. (rRNA represent around 1–10% of all RNA in EVs, while in a cell more than 90% of RNA are rRNA). (PDF) [file pone.0110443.s006.pdf]

Supporting information: S6

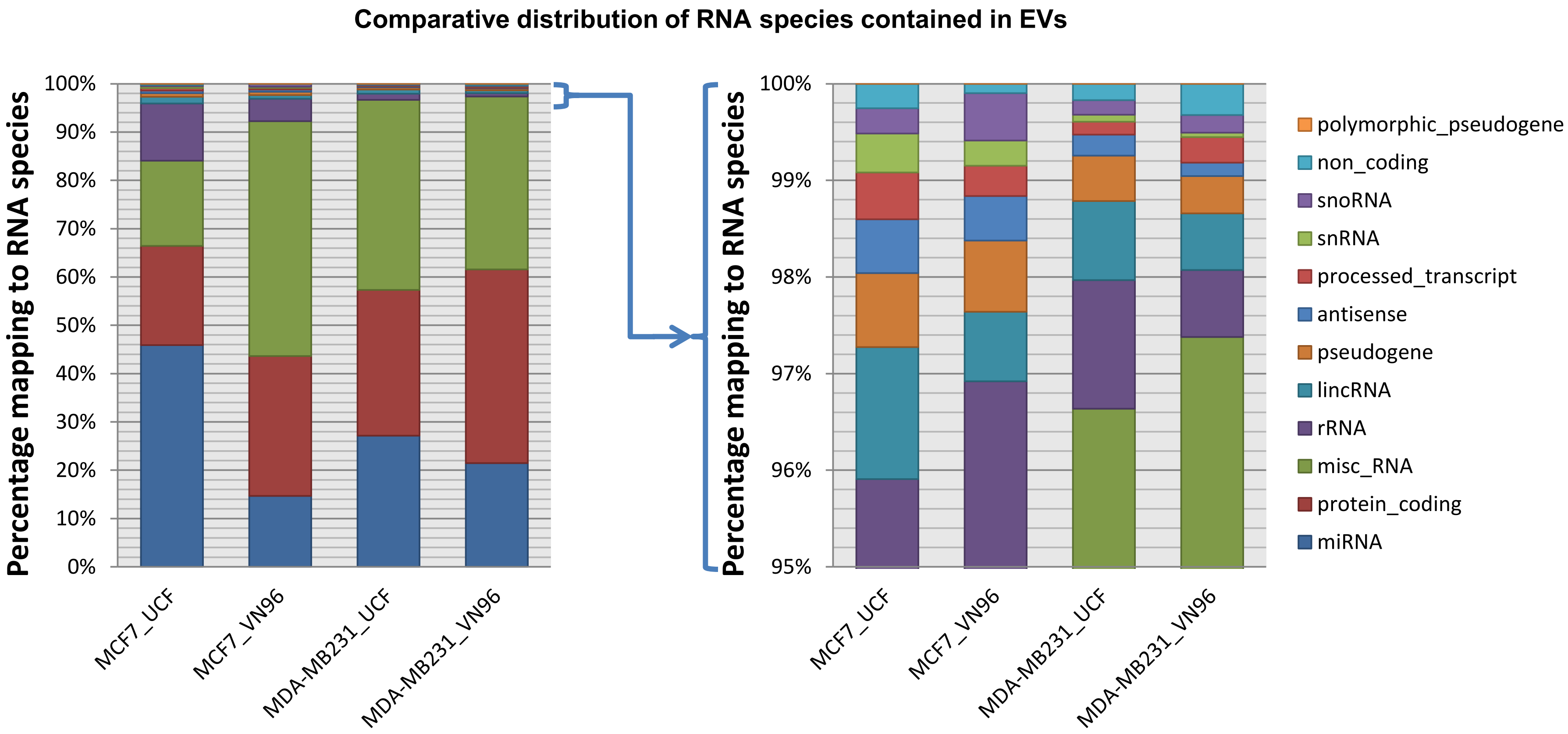

RNA species contained in EVs produced by breast cancer cell lines MCF-7 and MDA-MB-231 that were isolated by ultracentrifugation or the Vn96 peptide method. The right figure is an enlargement of the left figure in order to facilitate the visualization of less abundant RNA species. Proportions of RNA species are similar between isolation methods used. We also observed an enrichment of some RNA species in EVs compared to RNA species contained in the cell. (rRNA represent around 1-10% of all RNA in EVs, while in a cell more than 90% of RNA are rRNA).
